# Population-based physical activity promotion with a focus on health equity: a review of reviews

**DOI:** 10.1186/s12939-023-01834-5

**Published:** 2023-01-26

**Authors:** Simone Kohler, Lea Dippon, Natalie Helsper, Alfred Rütten, Karim Abu-Omar, Leonie Birkholz, Klaus Pfeifer, Philipp Weber, Jana Semrau

**Affiliations:** Department of Sport Science and Sport, Friedrich-Alexander University Erlangen Nuremberg, Gebbertstr, 123B, 91058 Erlangen, Germany

**Keywords:** Physical activity, Health promotion, Population-based approaches, Health equity, Social disadvantages

## Abstract

**Background:**

The extent to which people are physically active is dependent upon social gradients. Numerous studies have shown that especially people with social disadvantages do not meet the physical activity (PA) recommendations. A promising strategy to alleviate this issue are approaches that promote PA in the general population. In addition, several researchers have raised concerns that population-based health interventions may increase health inequities. The aim of the current review of reviews was therefore to identify successful population-based PA promotion approaches with a particular focus on health equity.

**Methods:**

Six electronic databases were examined for systematic reviews on population-based PA promotion for the period 2015 to 2021. A reference list and grey literature search were also conducted. Two independent reviewers used inclusion/exclusion criteria to screen titles and abstracts of the potentially relevant literature and conducted a quality assessment for each identified review. All included reviews of population-based approaches for PA promotion with a focus on disadvantaged populations and/or health equity were narratively summarized.

**Results:**

Our search resulted in 4,411 hits. After a systematic review process, six reviews met the inclusion criteria and were included after they were all rated as high quality. We identified that mass-media campaigns, point-of-decision prompts, environmental approaches, policy approaches, and community-based multi-component approaches can promote PA in the general population. Across populations with social disadvantages mass-media campaigns, point-of-decision prompts and policy approaches are likely to be effective as long as they are tailored. Regarding environmental approaches, the results are inconsistent. None of the reviews on community-based multi-component approaches provided evidence on health equity.

**Conclusion:**

There are several effective approaches to promote PA in the general population but evidence regarding health equity is still sparse. Future studies should therefore pay more attention to this missing focus. Furthermore, there is a lack of evidence regarding the type of tailoring and the long-term impact of population-based approaches to PA promotion. However, this requires appropriate funding programmes, complex study designs and evaluation methods.

**Supplementary Information:**

The online version contains supplementary material available at 10.1186/s12939-023-01834-5.

## Abstract (de)

**Hintergrund:**

Das Ausmaß in dem Menschen körperlich aktiv sind hängt vom sozialen Gradienten ab. Zahlreiche Studien haben gezeigt, dass insbesondere Menschen mit sozialer Benachteiligung die Empfehlungen für Bewegung nicht umsetzen. Bevölkerungsbezogene Maßnahmen sind geeignet, um alle Menschen zu erreichen, gleichzeitig gibt es wissenschaftliche Hinweise dafür, dass dadurch gesundheitliche Ungleichheiten verstärkt werden können. Ziel dieses Umbrella Reviews war es daher, wirksame bevölkerungsbezogene Ansätze zur Förderung der körperlichen Aktivität mit besonderem Fokus auf gesundheitliche Chancengleichheit zu identifizieren.

**Methoden:**

Mittels sechs elektronischen Wissenschaftsdatenbanken wurde für den Zeitraum von 2015 bis 2021 nach systematischen Reviews zur bevölkerungsbezogenen Bewegungsförderung gesucht.

Darüber hinaus wurde auch eine Handsuche in den Referenzlisten gefundener Artikel sowie eine Suche nach grauer Literatur durchgeführt. Zwei unabhängige Gutachter:innen überprüften anhand von Ein- und Ausschlusskriterien die Titel und Abstracts der potenziell relevanten Literatur und führten für jedes eingeschlossene Review eine Qualitätsbewertung durch. Die Ergebnisse der identifizieren Übersichtsarbeiten zu bevölkerungsbezogenen Ansätzen zur Bewegungsförderung mit Fokus auf Menschen mit sozialer Benachteiligung und/oder gesundheitliche Chancengleichheit wurden narrativ zusammengefasst.

**Ergebnisse:**

Insgesamt wurden 4.411 Reviews identifiziert. Nach einer systematischen Überprüfung, erfüllten sechs Reviews die Einschlusskriterien und wurden eingeschlossen, nachdem sie alle als qualitativ hochwertig eingestuft worden waren. Massenmediale Kampagnen, motivationale Entscheidungshilfen, umweltbezogene Ansätze, politikbezogene Ansätze und gemeindebasierte Mehrkomponentenansätze können das Bewegungsverhalten in der Allgemeinbevölkerung fördern. Für Bevölkerungsgruppen mit sozialer Benachteiligung liegt Evidenz vor, dass massenmediale Kampagnen, motivationale Entscheidungshilfen sowie politikbezogene Ansätze wirksam sind, sofern diese bedarfsgerecht (tailored) angepasst werden. Über umweltbezogene Ansätze wurden widersprüchliche Ergebnisse berichtet. Zu gemeindebasierten Mehrkomponentenansätzen fehlen bislang Ergebnisse hinsichtlich der Verbesserung von gesundheitlicher Chancengleichheit.

**Schlussfolgerung:**

Es wurden mehrere wirksame Ansätze zur Bewegungsförderung der Allgemeinbevölkerung identifiziert, jedoch sind Erkenntnisse über den Einfluss auf die gesundheitliche Chancengleichheit gering. Künftige Studien sollten daher diesem fehlenden Fokus mehr Aufmerksamkeit widmen. Des Weiteren mangelt es an Wissen über die Art der bedarfsgerechten Anpassung (Tailoring) sowie die langfristigen Auswirkungen bevölkerungsbezogener Ansätze zur Bewegungsförderung. Dies erfordert geeignete Finanzierungsprogramme, komplexe Studiendesigns und Evalierungsmethoden.

## Introduction

Although there has been evidence for the health benefits of physical activity (PA) since the 1950s [1], the global pandemic of physical inactivity is still ongoing. Furthermore, the risk of physical inactivity is unequally distributed: in industrialized countries people with social disadvantages are more often insufficiently physically active than those without [2–8]. For example, a recent secondary data analysis of 13 German cross-sectional studies shows that social disadvantages such as high age, low income, low levels of education, or a migrant background contribute to differences in several PA domains e.g. sports and vigorous PA [9]. Social disadvantage refers to the unfavourable social, economic, or political conditions that individuals or certain population groups systematically experience because of their relative position in the social hierarchy [10]. This means that people with a social disadvantage possess a restricted ability to fully participate in social processes because of limited or non-existent access to specific goods or resources. Furthermore, this contributes to health inequities with reduced opportunities for attaining the full health potential as well as higher mortality rates [11–13]. Due to these complex interrelations, physical inactivity as well as health inequities are two connected public health threats. Moreover, the development, implementation and evaluation of approaches to promote PA without increasing health inequity remains a major challenge [14, 15]. A promising strategy to attenuate the inactivity pandemic are approaches that promote PA in the general population [14, 16–19]. On the political level, international organizations also underline population-based approaches as an essential element for PA promotion. The ‘Eight Investments That Work For Physical Activity’ published by the International Society for PA and Health (ISPAH) [20], the Global PA Action Plan [21] or the WHO’s PA Strategy for the European Region 2016–2025 [22] are such examples.

However, some reviews point out that population-based approaches potentially increase the risk of health inequities. This is especially the case if they disproportionately reach the population, which is not affected by social disadvantages [23–25]. Accordingly, population-based approaches are promising in PA promotion and in improving health equity, when they consider proportionate universalism (i.e. actions that are universal and appropriate to the degree of need as a solution to reduce health inequalities) and thus address the general population and at the same time focus on population groups with social disadvantages [26, 14, 23]. For the successful implementation of population-based PA promotion approaches the community setting, here defined as a geographical area, is of particular importance. It is the place where people are born, grow up, live, work and age [26] and where the general population as well as population groups with social disadvantages can be reached. Moreover, as highlighted in the Ottawa Charter, the community setting is a central field of action in health promotion since social determinants of health and therefore health equity can be influenced [27, 28].

As part of the development of the German Recommendations for PA and PA Promotion, a review of reviews was conducted to assess the effectiveness of population-based approaches for PA promotion [29, 16]. Thirty-one reviews were included and they showed moderate evidence for mass-media campaigns, point-of-decision prompts, policy and environmental approaches, and multi-component community-based approaches [29, 30]. Current reviews also examine evidence for various population-based approaches [31–34] to promote PA. The focus on health equity is rarely found in these reviews, but rather in reviews focusing on the effectiveness of PA promotion approaches on target groups with social disadvantages [35–39].

In 2016, when the German Recommendations for PA and PA Promotion were published, the research on effects of population-based PA promotion approaches considering health equity was not sufficiently developed [30, 29]. Only two of the 31 included reviews [24, 40] addressed health equity in population-based approaches for PA promotion. In the UK, the National Institute for Health and Care Excellence also reported in the updated NICE guideline [NG90] ‘Physical activity and the environment update ‘ gaps in the evidence for the effectiveness of several population-based approaches among different population groups including socioeconomic groups [41].

Against this background, we conducted a review of reviews to update the previous review which was part of the development of the German Recommendations for PA and PA Promotion [29, 16]. Our aim was to identify current evidence on the effectiveness of population-based PA promotion in the community with a particular focus on health equity.

## Methods

### Search strategy/identification of studies

The search strategy used for the development of the German Recommendations for PA and PA promotion [29] was replicated to identify additional reviews published from January 2015 until December 2021. The databases Pubmed, Scopus, PsycInfo/SPORTDiscus (via Ebscohost), ERIC and IBSS (via Proquest) were searched in January 2022 filtered only by publication date. The following search terms: “physical activity”, “intervention”, “evidence”, “effect”, “health” and “review”. Alternative terms (e.g. bike, biking, cycling, walking, active transport, human powered transport, sedentary, exercise, sport) were defined as well as MESH terms. In addition to an electronic database search, we also conducted a reference list search and a grey literature search.

### Inclusion and exclusion criteria/selection criteria

Eligibility for inclusion was also oriented on the procedure described by Abu-Omar et al. [29] comprising the following criteria: (1) the review contains empirical results from single studies; (2) the review includes interventions focused on the promotion of PA; (3) the review focuses on the efficacy, and/or effectiveness of interventions; (4) the review is written in English or German. As the focus of the present article is specific, the following inclusion criteria was added: (5) the review includes population-based PA promotion approaches for the general population with a particular focus on socially disadvantaged population groups and/or health equity. Populations with social disadvantages are often described as those with low income, low education, low social status (e.g. unskilled workers), or other social disadvantages (e.g. single parents, migrants with poor language knowledge) [35, 42]. We accepted the definition of social disadvantage or health equity employed by the authors of the included studies.

Reviews (1) that focus on individuals rather than the general population within the community, (2) without any focus on population groups with social disadvantages or health equity, (3) in which the term community is not defined as a geographical area, and (4) printed before 2015 or already considered by Rütten & Pfeifer [16] were excluded.

### Data extraction and synthesis

**Fig. 1:**
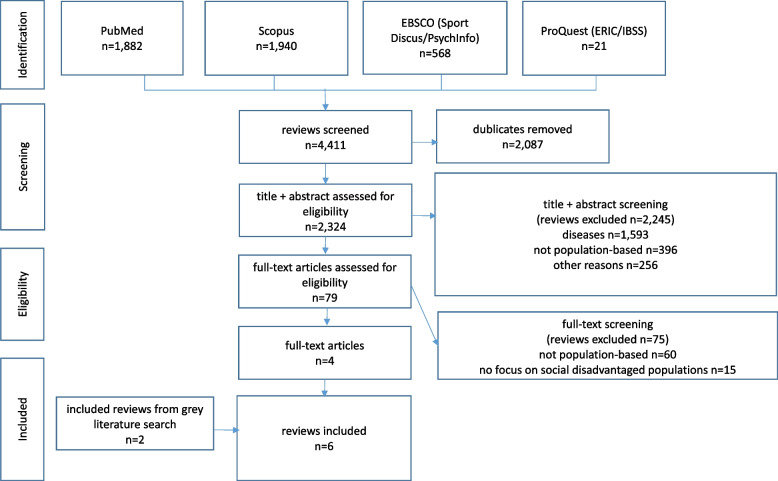
Literature search flow chart

We imported 4,411 search results into the bibliographic software program Citavi 6 and 2,087 through which duplicates were automatically deleted (Fig. 1) [43]. Three researchers (SK, LD, LB) independently screened 2,324 titles and abstracts. Seventy-nine abstracts met the inclusion criteria, and method. Cases in which the reviewers did not agree were discussed until consensus was reached. The main reasons for exclusion were that the approaches were not population-based (*n* = 60) or did not focus on disadvantaged populations (*n* = 15). Furthermore, one review [44] printed in 2015 was already included and discussed in the German Recommendations for PA and PA Promotion [16] and therefore excluded. Thus, four articles [6, 45–47] were included after the screening process, and two reviews [7, 48] were additionally obtained by grey literature search resulting in six articles in total.

### Quality assessment

For quality appraisal of the studies, two researchers (SK, JS) used the Scale for the Assessment of Narrative Review Articles (SANRA) assessing the quality of narrative review articles [49]. Studies were scored against six criteria (justification of the article’s importance for the readership; statement of concrete aims or formulation of questions; description of the literature search; referencing; scientific reasoning; appropriate presentation of data). The number of ratings was added to obtain a quality score. We classified reviews as adequate at a value between eight and twelve.

### Data synthesis

Two researchers (SK, JS) independently analysed the reviews by following the methodology proposed by Smith et al. [50]. For this, a table (Table 1) was developed presenting the following data: first author (publication year), title of the publication, type of review, number of included studies, type of PA promotion approach, main findings. The evidence on the impact, and/or the effectiveness, and/or the efficacy of population-based PA promotion interventions focusing on population groups with social disadvantages or health equity was narratively summarized and is presented in the results and discussion section. Although there is a substantive distinction in research between the terms impact, effectiveness, and efficacy, these terms are often used synonymously. Therefore, we have not made any amendments here, but have adopted the terminology of the authors.

**Table 1:** Reviews considering population-based PA promotion approaches for the general population with a particular focus on health equity or people with social disadvantages

Author	Title	Type of review	Type of data synthesis	Number of studies/reviews	Type of PA promotion approach	Main findings
Ball et al. 2015 [6]	Addressing the social determinants of inequities in physical activity and sedentary behaviours	Narrative Review	Narratively summarized	90 studies	environmental, policy, community-based multi-component, mass-media campaigns, point-of-decision prompts	- equitable promotion of PA promising for community-wide approaches; local and state governments to develop policies and practices; PA-friendly neighbourhood designs (including parks)
Cavill & Rutter, 2017 [7]	Health Equity Pilot Project (HEPP)—Evidence review: The impact of interventions and policies on SES differentials in physical activity	Umbrella Review	Narratively summarized + Expert opinion	6 reviews	environmental, policy, community-based multi-component	- urban regeneration programes, urban design and land use/transport policies, along with other attempts to revitalise the urban fabric and create more amenable and liveable conditions were effective to promote PA - no evidence of any differential impact on socioeconomic groups based on policy approaches - likely that those approaches reduce inequalities in health if implemented in areas of greatest need
Hunter et al. 2019 [45]	Environmental, health, wellbeing, social and equity effects of urban green space interventions: A meta-narrative evidence synthesis	Systematic Review	Meta-narrative evidence synthesis	38 studies	environmental	- supportive evidence for the use of certain urban green space (UGS) interventions for health, social and environmental benefits - strong evidence for park-based and greenway/trail interventions with a dual approach for park use and PA - too little evidence to draw firm conclusions on the impact of UGS interventions on a range of equity indicators
Smith et al. 2017 [46]	Systematic literature review of built environment effects on physical activity and active transport – an update and new findings on health equity	Systematic Review	Narratively summarized	28 studies	environmental	- positive effect of walkability components, provision of quality parks and playgrounds, and installation of or improvements in active transport infrastructure on active transport, PA, and visits or use of settings - some indication that infrastructure improvements may predominantly benefit socioeconomically advantaged groups
Thomas et al. 2018 [48]	A review of the impact of physical activity mass-media campaigns on low compared to high socioeconomic groups	Systematic Review	Narratively summarized	23 studies, 12 studies compared socioeconomic status differences	mass-media campaigns	- mostly equal or better impacts for the lowest SES (socioeconomic status) group compared to the highest SES group of PA mass-media campaigns - PA mass-media campaigns less frequently produced worse results for low SES groups
Olstad et al. 2016 [47]	Can policy ameliorate socioeconomic inequities in obesity and obesity-related behaviours? A systematic review of the impact of universal policies on adults and children	Systematic Review	Narratively summarized	36 studies	policy	- policies classified as agento-structural (change of structural aspects of environments but allow individual agency) or structural (change of the environmental context) and implemented at the macroenvironmental level or at the microenvironmental level did not negatively impact inequities in a range of PA behaviours (e.g. self-reported transportation-related PA, self-reported active travel, walking frequency, moderate to vigorous PA assessed via accelerometry)

## Results

### Overview

Overall, six reviews met the inclusion criteria (Table 1). These reviews were assessed for quality using the SANRA scale [49]. In this process, out of a possible total score of twelve, three reviews were rated as twelve, two as ten, and one as eight, allowing all six reviews to be rated as high quality and to be included in this review of reviews (Additional file 1: Appendix 1). Following the German Recommendations for PA and PA Promotion [16], we have structured our results in the following five types of population-based PA promotion approaches below.

### Mass-media campaigns

Two of the six identified reviews focused on mass-media campaigns.

A rapid review from Ball et al. [6] reported some evidence that supports the use of mediated approaches (i.e. PA promotion support delivered via media) in PA promotion amongst socially disadvantaged groups. The authors [6] found that approaches individually tailored to people with disadvantages have better chances of success based on two reviews. In general, the authors showed that mass-media campaigns alone do not effectively promote an increase in PA, but may be important for encouraging self-efficacy and/or knowledge related to PA without overly benefitting advantaged populations [6].

A further review [48] focused on PA mass-media campaigns and their impact on low compared to high socioeconomic status (SES) groups. The results span a period of 25 years and include 23 studies reporting on 17 PA mass-media campaigns. The authors stated that PA mass-media campaigns most often have similar or better impact on PA behaviour for people of low socioeconomic status compared to those with high socioeconomic status. They also added that “mass media campaigns need to be designed to maximize effectiveness for people from low SES groups” [48] and that ongoing evaluation should measure equity impacts.

### Point-of-decision prompts

One [6] of the six identified reviews examined the effectiveness of point-of-decision prompts. The authors identified on the basis of two reviews that point-of-decision prompts (e.g. promoting the use of stairs by providing information about health-related benefits) seem equally effective for promoting PA among adults across ethnic minority groups (if suitable tailored), and in diverse settings.

### Environmental approaches

Four reviews dealt with environmental approaches [6, 7, 45, 46].

Ball et al. [6] reported that the refurbishment of one public open space in a socioeconomically deprived area in Australia has shown a positive impact on PA among children and adults based on one study. However, one included longitudinal study showed that although a walking/cycling infrastructure after construction was accessible to a socioeconomically diverse population, it was more likely to be used by more socioeconomically advantaged adults.

Cavil et al. [7] found, based on four systematic reviews, environmental and transport approaches such as urban regeneration programes could increase PA, and that improving infrastructure for cycling could increase PA modestly, as well as infrastructure for walking could increase PA in the short-term. Overall, there was no evidence available for the differential impact of these interventions on health equity. They recommended providing high-quality physical environments for PA promotion without increasing health inequities, with a focus on revitalizing deprived communities and developing infrastructure that emphasizes walking and biking over motorized transportation.

One review [45] found strong evidence for park-based and greenway/trail interventions with a dual-approach to promote park use and PA. They also focused on a range of equity indicators like socioeconomic status, age or occupation. Their results are based on twenty studies in disadvantaged neighbourhoods and they suggest that there is insufficient information on the relations between urban green space interventions and a range of equity indicators.

Smith et al. [46] focused on the issue of built environment effects on PA considering health equity. They reported that infrastructure improvements such as enhancement of the neighbourhood walkability, quality of parks and playgrounds, and providing adequate active transport showed a consistent positive effect on PA, active transport and visits or use of settings. Four of the 28 included studies assessed differential effects of the built environment by ethnicity or socioeconomic status. While most analyses did not reveal statistically significant differences in effect by ethnicity or SES, two studies indicated that improvement in the built environment might predominantly benefit socioeconomically advantaged groups.

### Policy approaches

Three of the identified reviews dealt with policy approaches [6, 7, 47].

Ball et al. [6] noted, based on one example [51], that a policy for sharing PA facilities between the government, school, and community could improve opportunities for PA. Governance and policy interventions that consider such partnerships can meet the PA needs of the general population, including disadvantaged population groups. Ball et al. [6] further reported, based on three reviews, that transportation policies (e.g. improving infrastructure, providing incentives to encourage walking or cycling as active transport modes) are promising for effective and sustainable PA promotion in general. However, there is lacking evidence regarding the impact of transport policy approaches on PA among disadvantaged populations.

Ball et al. [6] and Cavil et al. [7] identified the same review [52] that focused on urban design, land-use and transport policies and practices to increase PA. This review reported no differential impacts on socioeconomic groups based on policy approaches. Moreover, the authors stated that given the diversity of population-groups included in this study, these results are likely to be applicable to diverse population groups, as long as interventions are tailored to the target population. However, the authors did not specify how the interventions should be tailored.

Another review [47] examined whether universal policies can ameliorate socioeconomic inequities in obesity and obesity-related behaviours like PA. Most of the seven included studies that assessed PA outcomes showed a neutral and some positive impact of policy on socioeconomic inequities in several PA outcomes (e.g. self-reported or objectively measured PA, walking frequency, active travel, transportation-related PA). The policy types ranged from structural (change in the environmental context) to agento-structural (change in the environmental context, but allow individual agency) policies implemented at the macroenvironmental level or at the microenvironmental level (size of the environment where the policy is implemented).

### Community-based multi-component approaches

Three reviews [45, 6, 7] reported about community-based multi-component approaches that combine structural components (environment and/or policy) and behavioural approaches.

Recommended by Ball et al. [6], multi-component, appropriately tailored whole-of-community campaigns are a potential intervention strategy. Based on six reviews they conclude that “large-scale, highly visible, multi-component campaigns involving multiple sectors and partnerships” [6] are appropriate for PA promotion but the effectiveness for population groups with disadvantages has not been well studied and results are inconsistent.

Hunter et al. [45] who focused on effects of urban green space interventions and less on community-based multi-component approaches also identified strong evidence for park use interventions to promote PA when employing a dual-approach (i.e. change in the environment combined with a marketing program). However, regarding the impact of the dual-approach on population groups with social disadvantages or health equity, the authors reported that no evidence was available. On the other hand, Ball et al. [6] reported that the combination of environmental and individual components has demonstrated effectiveness regarding PA promotion among diverse population groups, including disadvantaged population groups.

Cavil et al. [7] refer to a Cochrane Review [44] in which the authors noted that some studies with environmental approaches observed that more people walked. However, this review did not show that the multi-component community wide interventions increased PA in the population. Although none of the included studies there provided results regarding health inequity, it is noteworthy that 14 of the 25 studies conducted in high-income countries were implemented in disadvantaged or deprived communities.

## Discussion

The aim of this review of reviews was to provide an overview of the available evidence on the effectiveness of population-based PA promotion approaches in the community with a focus on health equity. In addition the results reported here represent an update for the chapter "Recommendations for Physical Activity Promotion for the General Population" of the German Recommendations for PA and PA Promotion published in 2016 [16, 29]. Since the last search in 2015, we identified six new reviews. Compared to only two reviews identified during the development of the German Recommendations with a search period of ≤ March 2015, this indicates a growing interest in the scientific community. Overall, our review of reviews showed that mass-media campaigns, point-of-decision prompts, environmental approaches, policy approaches, and community-based multi-component approaches can promote PA in the general population. However, the evidence regarding health equity is still sparse and future studies should assess the theoretical basis of these approaches, their differential impact including the potential negative and unintended consequences (e.g. stigma, gentrification) as well as the long-term impact on PA promotion and health equity.

Regarding mass-media campaigns [48], point of decision prompts [6] and policy approaches [6, 7] tailoring was mentioned several times as a critical criterion for effective and equitable implementation of population-based PA promotion approaches. However, most of the reviews provide little information about specific strategies of tailoring within such approaches based on the included studies. Tailored interventions can differ depending on the underlying paradigm [53]. Tailoring means, for example, an intervention tailored to specific characteristics of an individual or group to promote individual behavior change. Tailoring is also when community-based participatory action approaches that engage multiple actors in communities consider their needs and assets with the aim of promoting social change [53, 54]. Tailoring a PA intervention to specific characteristics of a person with social disadvantages may also unintentionally contribute to stigma [55], which was not discussed in the included reviews. Strategies that involve people with social disadvantages in the development, implementation and evaluation of population-based PA approaches and promote their empowerment seem to be therefore more promising [40, 16]. A concrete description of tailoring strategies within population-based PA promotion approaches that address the whole population and particularly consider people with social disadvantages is important to understand their long-term effectiveness as well as their opportunities and challenges and to scale them up successfully and sustainably.

Another finding was that evidence on the effectiveness of mass-media campaigns and and point of decision prompts used by itself is insufficient [6]. This supports the German Recommendations for PA and PA Promotion [16] which also recommend mass-media campaigns and point-of-decision prompts as part of a multi-component approach that integrates especially structural components (environment and policy) as well as context-based PA programs. Both, our current review of reviews as well as the German Recommendations were not able to provide any information about differential effects on health equity due to a lack of studies [16].

The results on environmental approaches [6, 7, 45, 46] in this review of reviews show a positive effect on PA for the general population. With respect on health equity, all reviews stated a lack of studies. Based on a limited number of studies two reviews focusing on environmental approaches indicate that populations with social disadvantages might also benefit from interventions implemented in socially deprived areas [6, 7]. However, it was also reported, that changes in the built environment might predominantly benefit socioeconomically advantaged groups [46, 6]. The implementation in deprived areas may have potentially negative consequences such as gentrification when changes in the neighbourhood might promote a transition towards a more privileged population [56]. Only one [45] of four included reviews discussed negative impacts of environmental approaches and undesirable effects on the population. Therefore, future research on the differential impact of environmental approaches on PA and the underlying positive but also negative mechanisms for health equity is essential to achieve the desired effectiveness.

Our review of reviews showed that policies for intersectoral partnerships, transportation policies, and land use policies are promising for the effective and sustainable promotion of PA in the general population [6, 7, 47]. This is not consistent with a recent review of reviews on effective policies for PA promotion, which was not included here, as it did not report differential effects on health equity [34]. Gelius et al. [34] found mixed evidence for the effectiveness of active travel policies and local transportation policies.

Regarding the impact of policy approaches on PA and health equity, it is not possible to draw a general conclusion. Our main finding for policy approaches on health equity reported by Olstadt et al. [47] showed that universal policies (i.e. those addressed at the general population) have mainly a neutral impact on socioeconomic inequities in several PA outcomes. This result for universal policies, based on a limited number of studies, is not in line with the inequality paradox stating that population-based approaches potentially increase health inequity by reaching mainly socially advantaged populations [23]. If universal policy approaches took into account a specific focus on populations with social disadvantages in the spirit of proportionate universalism [28], this could improve health equity in PA outcomes.

The evidence on community-based multi-component approaches based on the included reviews is also sparse [6, 7, 45]. Overall, community-based multi-component approaches are recommended in PA promotion, if structural components (environment and policy) and behavioural approaches are combined. Nevertheless, none of the three identified reviews was able to provide evidence on the impact on health equity.

The German Recommendations for PA and PA Promotion state that multi-component approaches should primarily use the mutual interaction of effective individual components [16]). In addition, to increase the knowledge about the synergistic effects between components and to gain a deeper understanding of such a complex approach in PA promotion a health equity perspective is urgently needed for future research. Further insights about the theoretical basis on multi-component approaches are also required.

In general, our research shows – despite an increase in publications in recent years – a lack of studies for population-based PA promotion approaches with a particular focus on population groups with social disadvantages. The need for more emphasis on this issue becomes evident upon closer examination of our results, considering we had to exclude 75 reviews in the full-text screening because they only focused on individual interventions to behaviour change and did not consider population as a whole. The reason for the lack of studies highlighted here could be the complexity of population-based PA promotion approaches with a focus on health equity as they require multiple efforts in various sectors, the involvement of multiple stakeholders und a lot of resources [19]. Individual behaviour change interventions even with or without a focus on health equity, are comparatively easier to implement and require fewer human and financial resources. Consequently, less elaborate or resource-intensive individual behavior change interventions may be more attractive to practitioners and implemented more frequently. Though, evidence on the long-term effectiveness of individual behavior change interventions to promote PA and to reduce health inequities is lacking [6, 29, 35, 57].

In addition to the challenges in implementing such complex population-based approaches, the evaluation of those also represent another difficulty [58]. Thus, in contrast to established standards of evidence-based medicine and the use of randomized, controlled trials as gold standard, there are no standard evaluation methods available for complex population-based PA promotion approaches [58]. Nonetheless, it is recommended to evaluate all phases [59] of the implementation and to assess more closely the input, process, output, outcome and impact of PA promotion interventions [60]. Although such an evaluation requires high resources, it is worthwhile investing them to better understand the complex interrelationships and interactions of population-based PA promotion approaches and health equity. Qualitative as well as mixed-methods evidence syntheses provide an important contribution to this research.

Without a health equity focus to policy, practice and research, health inequities will not be effectively reduced [61] through population-based PA promotion approaches. Rather, if health equity is neglected, our review of reviews shows that there is a risk that such approaches will not reach those who would need it most, potentially widening the health inequity gap. The missing evidence regarding health equity also becomes apparent when the perspective is not limited to PA promotion, but broadened to consider health promotion interventions under the principle of proportionate universalism [58, 28]. Therefore, in the development, implementation and evaluation of population-based PA promotion approaches the collective action with actors from politics, practice and research with a stronger focus on health equity is an essential strategy to consider population groups with social disadvantages and to sustainably promote PA and to reduce health inequities [45].

### Strength and limitations

There are some limitations concerning the present review of reviews. One issue for discussion is the causality between population-based PA promotion approaches, the corresponding PA outcomes and health equity. Since randomized, controlled trials are often not suitable to assess the effectiveness of these complex approaches, it was not possible to draw conclusions about causal effects, but only to observe associations. Furthermore, our review of reviews was limited by the information given in the included reviews and the reported limitations. Nevertheless, where possible, we also used the information from the primary studies cited, but vague descriptions often made it difficult to discern precise associations. One often reported limitation in the included reviews was that most studies were conducted in high-income countries, so statements about the effectiveness of population-based PA promotion approaches in low- and middle-income countries cannot be made. Another reported limitation was the heterogeneity concerning definitions and description of social disadvantages, intervention approaches, study designs, inclusion criteria, evaluation approaches and outcome measures, which limits the strength of findings and the derivation of conclusions regarding the impact of population-based PA approaches on PA and health equity. Moreover, many included studies in the reviews had rather short follow-up periods, which limits the evidence base regarding the long-term effectiveness of such approaches. Finally, population-based PA promotion approaches with a focus on health equity, are complex interventions in complex systems and an emphasis on the isolation of effects of specific features of these approaches may limit the understanding of the aggregated benefits of them.

A strength of this review of reviews is the application of a comprehensive method to capture a broad range of population-based PA promotion approaches. In addition, we did not limit the search from the beginning by including population-based approaches and health equity in the search terms, but rather screened title, abstract and full texts for population-based approaches and aspects of health equity to decide whether they are suitable to our research question. Furthermore, following best practices, we adopted a systematic approach to literature identification and screening, data extraction, and quality assessment of the relevant reviews.

## Conclusion

This article presents an overview of the recent evidence on the effectiveness of population-based PA promotion approaches with a focus on health equity.

Mass-media campaigns, point-of-decision prompts, environmental approaches, policy approaches, and community-based multi-component approaches can promote PA in the general population.

Although we identified six new reviews since the last search in 2015 the evidence regarding health equity is still inconclusive. As long as they are tailored to the needs of the population groups, mass-media campaigns, point-of-decision prompts and policy approaches seem to be equally effective for PA promotion across population groups with social disadvantages. How the interventions should be tailored in detail remains unclear. For environmental approaches, the results are not consistent and it is not answered whether people with social disadvantages in particular benefit from interventions in deprived areas. Regarding community-based multi-component approaches, none of the reviews could show the impact of these complex approaches on health equity.

However, when interpreting the findings, the heterogeneous body of evidence and a lack of studies evaluating population-based PA promotion approaches with a focus on health equity should be considered.

Future research should consider the complex nature of population-based PA promotion approaches and assess the theoretical basis of these approaches, their differential impact including potential negative and unintended consequences (e.g. stigma, gentrification) as well as the long-term impact on PA promotion and health equity. This requires sufficient funding schemes, appropriate study designs, and evaluation methods to assess these complex approaches. In this way, we can deepen our knowledge and thus make a substantial contribution to promoting PA and improving health equity.

## Supplementary Information

**Additional file 1:** **Appendix 1.** Scale for the Assessment ofNarrative Review Articles (SANRA) assessing the quality of narrative reviewarticles.

## Data Availability

Not applicable.

## Abbreviations

PA: Physical activity
ISPAH: International Society for PA and Health
