# Supplementary material for: Population-based physical activity promotion with a focus on health equity: a review of reviews
Source: Int J Equity Health. 2023 Jan 26;22:18. doi: 10.1186/s12939-023-01834-5 (PMC9878967; doi:10.1186/s12939-023-01834-5)
Supplement: Supplementary file 1 — Additional file 1: Appendix 1. Scale for the Assessment ofNarrative Review Articles (SANRA) assessing the quality of narrative reviewarticles. [file 12939_2023_1834_MOESM1_ESM.docx]

| **Author** | **Justification of the article’s importance for the readership** | | | **Statement of concrete aims or formulation of questions** | | | **Description of the literature search** | | | **Referencing** | | | **Scientific reasoning e.g., incorporation of appropriate evidence, such as RCTs in clinical medicine)** | | | **Appropriate presentation of data (e.g., absolute vs relative risk; effect sizes without confidence intervals)** | | | **total** |
| --- | --- | --- | --- | --- | --- | --- | --- | --- | --- | --- | --- | --- | --- | --- | --- | --- | --- | --- | --- |
|  | The importance is not justified | The importance is alluded to, but not explicitly justified | The importance is explicitly justified | No aims or questions are formulated | Aims are formulated generally but not concretely or in terms of clear questions | One or more concrete aims or questions are formulated | The search strategy is not presented | The literature search is described briefly | The literature search is described in detail, including search terms and inclusion criteria | Key statements are not supported by references | The referencing of key statements is inconsistent | Key statements are supported by references | The article’s point is not based on appropriate arguments | Appropriate evidence is introduced selectively | Appropriate evidence is generally present | Data are presented inadequately | Data are often not presented in the most appropriate way. | Relevant outcome data are generally presented appropriately |  |
| Ball et al. 2015 |  |  | 2 |  |  | 2 |  |  | 2 |  |  | 2 |  | 1 |  |  | 1 |  | 10 |
| Cavill & Rutter, 2018 |  |  | 2 |  |  | 2 |  | 1 |  |  |  | 2 | 0 |  |  |  | 1 |  | 8 |
| Hunter et al. 2019 |  |  | 2 |  |  | 2 |  |  | 2 |  |  | 2 |  |  | 2 |  |  | 2 | 12 |
| Smith et al. 2017 |  |  | 2 |  |  | 2 |  |  | 2 |  |  | 2 |  |  | 2 |  |  | 2 | 12 |
| Thomas et al. 2018 |  |  | 2 |  |  | 2 |  |  | 2 |  |  | 2 |  | 1 |  |  | 1 |  | 10 |
| Olstad et al. 2016 |  |  | 2 |  |  | 2 |  |  | 2 |  |  | 2 |  |  | 2 |  |  | 2 | 12 |
